# Factors influencing medical and nursing students’ willingness to care for COVID-19 patients in South Korea: a cross-sectional study

**DOI:** 10.1186/s12909-022-03229-6

**Published:** 2022-03-08

**Authors:** Eun A Kim, Hae Ran Kim, Boyoung Kim

**Affiliations:** 1 Department of Nursing, Honam University, 417 Eodeung-daero, Gwangsan-gu, Gwangju, 62399 Republic of Korea; 2 Department of Nursing, College of Medicine, Chosun University, 309 Pilmun-daero, Dong-gu, Gwangju, 61452 Republic of Korea; 3 College of Nursing, Chonnam National University, 160 Baekseo-ro, Dong-gu, Gwangju, Republic of Korea

**Keywords:** Attitudes, COVID-19, Healthcare, Korea, Medical students, Nursing students, Willingness to care

## Abstract

**Background:**

The COVID-19 pandemic has threatened the stability and adequacy of the global healthcare system. In this situation, authorities have considered engaging medical and nursing students to address the shortage of frontline healthcare workers. This study investigated the effect of COVID-19-related knowledge, attitudes, and preventive behaviors on medical and nursing students’ willingness to care for COVID-19 patients.

**Methods:**

Medical and nursing students from universities in two regions of South Korea participated in this cross-sectional study. A total of 330 questionnaires were distributed; of them, 99.3% were collected, and 315 questionnaires were analyzed. Data were collected on participants’ general characteristics, COVID-19-related knowledge, attitudes, and preventive behaviors, and willingness to care for COVID-19 patients.

**Results:**

In total, 66.3% of the participants were willing to care for patients during the COVID-19 pandemic. Students in higher grades and those with more clinical practice experience showed higher levels of willingness to care. Specifically, willingness to care was correlated with the senior year (OR = 3.58, 95% CI = 1.24 − 10.37, *p* = .019), a high level of COVID-19-related knowledge (OR = 1.03, 95% CI = 1.00 − 1.05, *p* = .041), an optimistic attitude that COVID-19 can be overcome (OR = 1.63, 95% CI = 1.24 − 2.14, *p* < .001), and increased engagement in infection prevention behaviors (OR = 1.41, 95% CI = 1.16 − 1.71, *p* < .001).

**Conclusions:**

The findings indicated that a high level of knowledge regarding the COVID-19 pandemic, having an optimistic attitude, and engaging in preventive behaviors affected students’ willingness to care for COVID-19 patients. Integrating disaster preparedness courses in the early years of their curriculum could increase future healthcare providers’ willingness to care for patients.

## Background

The COVID-19 pandemic threatens public health on a global scale due to its rapid and persistent human-to-human transmission [1]. Although the case fatality ratio of COVID-19 has been low [2], the disease has caused more deaths than the severe acute respiratory syndrome (SARS) outbreak in 2003 and the Middle East respiratory syndrome (MERS) outbreak between 2012 and 2019 combined [3]. Since January 2020, the number of COVID-19 cases across South Korea have been rising rapidly and steadily [4]. Wearing a mask, practicing social distancing, and self-isolation have become common measures to prevent infection. The pandemic has also had a significant effect on people’s daily lives due to the closure of schools and workplaces [5] and a shift to the online mode of learning and working.

Healthcare providers, especially physicians and nurses, are at serious risk of infection and even death because they are liable to come into contact with asymptomatic or even critically ill COVID-19 patients [6]. Regardless of the occupational risks, physicians and nurses play a vital role in the proper functioning of the healthcare system and effective response to pandemic situations [7]. The unmitigated rise in cases of infection and mortality rates may affect medical and nursing students’ willingness to care for COVID-19 patients. In a recent study on soon-to-graduate nursing students in South Korea, only 2 of 58 participants were firmly willing to enter the nursing profession after graduating, owing to the impact of COVID-19 [8]. A study on medical students in Saudi Arabia found that only 34% of the participants were willing to be frontline workers during the pandemic [9], while another study on Chinese medical students reported that just 18% of the participants were willing to work in the field of infectious diseases such as COVID-19 [10]. Students’ low levels of willingness to care for patients amid a disaster can lead to fluctuations and shortages in the number of healthcare providers, thus worsening the pandemic situation [11].

Medical and nursing students, who are expected to play an important role in the healthcare workforce, gain technical knowledge as well as learn the necessary attitudes [12] and behaviors [13] during their education and training. Thus, these students form opinions regarding their willingness to care for patients in a crisis, such as the COVID-19 pandemic, during their training period [14]. Recent studies reported that students with a broad range of essential clinical skills and knowledge regarding infectious diseases were highly willing to participate as healthcare providers during the COVID-19 pandemic [9, 15, 16]. A study on final-year nursing students appointed to provide healthcare services during the COVID-19 pandemic found that the students’ willingness to participate may decrease if they have negative attitudes and feel that they themselves or their families may be in danger. However, experience in infection prevention practices increased students’ responsibility when caring for patients during the pandemic [17].

The rapid spread of COVID-19 and the resulting increase in the number of patients has caused overloading in healthcare systems, leading to a serious shortage of healthcare providers [18]. Gaining an understanding of medical and nursing students’ willingness to fulfill their professional duties by participating as frontline healthcare workers during the COVID-19 pandemic can help develop future disaster response strategies [19]. In South Korea, to date, there has been limited research regarding the relationship between COVID-19-related knowledge, attitudes, and preventive behaviors of medical and nursing students and their willingness to care for patients during the COVID-19 pandemic. Therefore, this study investigated medical and nursing students’ willingness to care for COVID-19 patients, as well as the factors influencing their willingness, in South Korea. Specifically, it investigated the effect of students’ COVID-19-related knowledge, attitudes, and preventive behaviors on their willingness to care for COVID-19 patients.

## Methods

### Design and participants

This study employed a cross-sectional descriptive research design and was conducted in two stages. First, a pilot study was conducted to verify the validity and reliability of the measurement tool [20]. As willingness to care for COVID-19 patients was measured using a single questionnaire item, only the content validity was verified. Then, the main study was conducted to investigate the factors that affect the willingness of medical and nursing students to care for COVID-19 patients; the questionnaire included participants’ general characteristics, willingness to care for COVID-19 patients (one item), COVID-19-related knowledge (eight items), attitudes (two items), and preventive behaviors (nine items).

Students from four universities, located in two regions of South Korea, were recruited using convenience sampling. The participants comprised students in the preclinical (first and second year) and clinical years (first and second year) of medical school, as well as students from the nursing department (first to fourth year). Previous research was consulted to ensure the selection of occupational groups that are most exposed to COVID-19 [21]. Studies with a similar research design were used as reference [22, 23], and the G*Power 3.1.9 program was used to calculate the sample size. The minimum sample size required to perform a logistic regression analysis was calculated to be 298, with the following conditions: a two-tailed test, significance level at ⍺ = 0.05, odds ratio = 1.70, null hypothesis probability of H_0_ = 0.2, and a power of 0.95. Accounting for a dropout rate of 10%, a total of 330 questionnaires were distributed; of these, 99.3% were collected, and a total of 315 questionnaires were analyzed.

### Procedure

This study was performed in line with the principles of the Declaration of Helsinki and was approved by the University Institutional Review Board in South Korea. Data were collected in June 2020; Google Forms was used to distribute and collect the questionnaires. To conduct the survey, the purpose of this study was explained to professors in the four universities. After obtaining their consent, an identical link was sent to each of the professors, who then explained the study’s purpose to their students and distributed the link. Participants were instructed to fill out a self-report questionnaire after providing informed consent for the study online. The students who completed the questionnaire were given a token of appreciation (an online coupon).

### Variables

#### General characteristics

Previous studies were taken into consideration [9, 20, 24, 25] to collect data on factors that could affect willingness to care for COVID-19 patients, including participants’ sex, academic major, grade level, religion, subjective health status, and clinical practice experiences during the COVID-19 pandemic. Students in the first and second year of medical school, and first and second year of the nursing department were classified as “sophomores.” Students in the third and fourth year of medical school and third year of the nursing department were classified as “juniors.” Finally, students in the fifth and sixth years of medical school and fourth year of the nursing department were classified as “seniors.”

#### Willingness to care for COVID-19 patients

A questionnaire item modified from a previous study [23] was used to measure the participants’ willingness to care for COVID-19 patients. Participants could only respond with “I do not agree,” “I do not know,” or “I agree” to the following statement: I am willing to care for patients who have been or may have been infected with COVID-19 on the front lines. A response of “I agree” was classified as “willingness,” while the other responses were classified as “unwillingness.”

#### COVID-19-related knowledge

The measurement scale in Taghrir et al. [26] was used to measure students’ knowledge, after modifying it to fit the situation in South Korea. The eight-item questionnaire includes items on public preventive measures (three items), basic etiology and diagnoses (three items), and COVID-19 symptoms and treatments (two items). Participants could respond to each item with “No,” “I do not know,” or “Yes.” A correct response was given 1 point, whereas the other responses were given 0 points. Scores were calculated by adding up the points and converting them to a 100-point scale. The total score ranged from 0–100 points; higher scores indicated higher levels of knowledge regarding COVID-19. When the measurement tool was developed, its reliability was demonstrated with a Cronbach’s α of 0.80. This study used the Kuder–Richardson Formula 20 (KR-20) to analyze the tool—the overall reliability was 0.81.

#### COVID-19-related attitudes

The questionnaire employed by Zhong et al. [21] was used to assess students’ attitudes; it was modified to fit the situation in South Korea. It consists of two items regarding the prevention and control of the spread of COVID-19 and the confidence that the battle against COVID-19 can be won. Participants had to respond to each item with “I do not agree” (0 points), “I do not know” (1 point), or “I agree” (2 points). The total score ranged from 0–4 points, with higher scores indicating an optimistic attitude that COVID-19 can be overcome. The reliability of the scale was demonstrated by a Cronbach’s α of 0.78 in this study.

#### Preventive behaviors

A measurement tool employed in previous studies was used after modification to fit the situation in South Korea [26, 27]. The questionnaire investigated behavior in public places (four items), personal hygiene and sharing behaviors (three items), and self-isolating behaviors (two items). Participants responded to each item with either “no” (0 points) or “yes” (1 point). The total score ranged from 0–9 points, with higher scores signifying increased engagement in COVID-19 infection prevention behaviors.

### Validity and reliability

Prior to the study, six experts were invited to review the measurement tools, including professors of medicine, infectious diseases, and nursing education. To clarify the acceptability of the questionnaire, a preliminary survey was conducted on 35 students, who reported that the items were clear and easy to understand [20]. As a result of these reviews, the content validity index of the questionnaire was deemed appropriate.

### Data analysis

The data were analyzed using IBM SPSS Statistics for Windows, version 24 (IBM Corp., Armonk, NY, USA). The independent t-test and χ^2^ test were used to perform a univariate analysis on the differences between students’ willingness to care for COVID-19 patients based on their general characteristics, COVID-19-related knowledge, attitudes, and preventive behaviors. Additionally, a binary logistic regression analysis was conducted to identify the factors that affect willingness to care for COVID-19 patients. Only items with *p* < 0.05 in the univariate analysis were input into the regression analysis, and the Hosmer–Lemeshow test was used to determine the goodness-of-fit of the model.

## Results

### Differences in willingness to care for COVID-19 patients based on participants’ general characteristics

Of the medical and nursing students who participated in this study, 66.3% (*n* = 209) expressed a willingness to participate in COVID-19 patient care, and 33.7% (*n* = 106) responded that they were unwilling or unsure. There was a significant difference in the willingness to care for COVID-19 patients according to grade level/academic year (χ^2^ = 21.27, *p* < 0.001) and clinical practice experience during the COVID-19 pandemic (χ^2^ = 15.10, *p* < 0.001) (Table 1). For instance, 81.1%, 68.1%, and 48.9% of the seniors, juniors, and sophomores, respectively, responded that they were willing to participate in COVID-19 patient care. Additionally, 75.7% and 54.9% of the participants with and without clinical practice experience, respectively, responded that they were willing to participate in the care of COVID-19 patients (Table 1).

**Table 1:** Willingness to care for COVID-19 patients according to general characteristics (*N* = 315)

Variables	Total (n, %)	Willingness to care for COVID-19 patients (*n* = 209)	Unwillingness to care for COVID-19 patients (*n* = 106)	χ2	*p*
Gender				0.03	0.863
Male	102(32.4)	67(65.7)	35(34.3)		
Female	213(67.6)	142(66.7)	71(33.3)		
Grade				21.27	< 0.001
Sophomore	90(28.6)	44(48.9)	46(43.4)		
Junior	135(42.8)	92(68.1)	43(31.9)		
Senior	90(28.6)	73(81.1)	17(18.9)		
Major				0.07	0.796
Nursing	193(61.3)	127(65.8)	66(34.2)		
Medicine	122(38.7)	82(67.2)	40(32.8)		
Religion				2.24	0.134
Yes	113(35.9)	81(71.7)	32(28.3)		
No	202(64.1)	128(63.4)	74(36.6)		
Subjective health status				2.46	0.292
Poor	19(6.0)	14(73.7)	5(26.3)		
Moderate	107(34.0)	65(60.7)	42(39.3)		
Good	189(60.0)	130(68.8)	59(31.2)		
Clinical practice experiences during the COVID-19				15.10	< 0.001
Yes	173(54.9)	131(75.7)	42(24.3)		
No	142(45.1)	78(54.9)	64(45.1)		

*COVID-19* Coronavirus Disease-19, Data are expressed as n (%)

### The association between COVID-19-related knowledge, attitudes, and preventive behaviors and willingness to care for COVID-19 patients

The mean score for COVID-19-related knowledge was statistically and significantly higher in the willingness group (90.61) compared with the unwillingness group (86.9; t = 2.86, *p* = 0.005). Similarly, the willingness group scored significantly higher on COVID-19-related attitudes compared with the unwillingness group (3.44 vs. 2.88; t = 4.39, *p* < 0.001). Furthermore, the willingness group obtained higher scores for COVID-19-related preventive behaviors compared with the unwillingness group (8.34 vs. 7.65; t = 4.39, *p* < 0.001) (Table 2).

**Table 2:** Knowledge, attitude, and preventive behaviors between the willingness group and unwillingness group (*N* = 315)

Variables	Total	Willingness to care for COVID-19 patients (*n* = 209)	Unwillingness to care for COVID-19 patients (*n* = 106)	t	*p*
Knowledge	89.37 ± 10.97	90.61 ± 9.13	86.91 ± 13.63	2.86	0.005
Attitude	3.25 ± 0.98	3.44 ± 0.80	2.88 ± 1.18	4.39	< 0.001
Preventive behaviors	8.11 ± 1.35	8.34 ± 1.03	7.65 ± 1.73	3.77	< 0.001

*COVID-19* Coronavirus Disease-19, Data are expressed as mean ​ ± ​standard deviation (SD)

### Factors influencing willingness to care for COVID-19 patients

The variables that showed a significant difference during the univariate analysis were general characteristics (grade level and clinical practice experience) and COVID-19-related knowledge, attitudes, and preventive behaviors. A binary logistic regression analysis was performed with the abovementioned characteristics as the independent variables and the willingness and unwillingness groups as the dependent variables. The results indicated that participants’ willingness to care for COVID-19 patients was affected by their grade level and COVID-19-related knowledge, attitudes, and preventive behaviors. Specifically, willingness to care was related to the senior year (OR = 3.58, 95% CI = 1.24 − 10.37, *p* = 0.019), a high level of COVID-19-related knowledge (OR = 1.03, 95% CI = 1.00 − 1.05, *p* = 0.041), an optimistic attitude that COVID-19 can be overcome (OR = 1.63, 95% CI = 1.24 − 2.14, *p* < 0.001), and increased engagement in infection prevention behaviors (OR = 1.41, 95% CI = 1.16 − 1.71, *p* < 0.001). Furthermore, the regression model significantly predicted the factors that affect participants’ willingness to care for COVID-19 patients (χ^2^ = 54.64, *p* < 0.001). The Nagelkerke coefficient of determination demonstrated an explanatory power of 22.1%. The classification accuracy of the model was found to be 70.2%. The results of the Hosmer–Lemeshow goodness-of-fit test did not reject the null hypothesis that there was no difference between the observed and predicted values (χ^2^ = 18.01, *p* = 0.121), Thus, the model was determined to be suitable (Table 3).

**Table 3:** Factors influencing willingness to care for COVID-19 patients (*N* = 315)

Variables	B	SE	*p*	OR	95% CI
Grade					
Sophomore (reference)					
Junior	0.42	0.39	0.287	1.52	0.70–3.29
Senior	1.28	0.54	0.019	3.58	1.24–10.37
Clinical practice experiences					
Yes (Reference)					
No	-0.08	0.40	0.853	0.93	0.42–2.05
Knowledge	0.03	0.01	0.041	1.03	1.00–1.05
Attitude	0.49	0.14	< 0.001	1.63	1.24–2.14
Preventive behaviors	0.35	1.00	< 0.001	1.41	1.16–1.71

*COVID-19* Coronavirus Disease-19, *SE* Standard error, *OR* Odds ratio, *CI* Confidence interval

## Discussion

Of the medical and nursing students who participated in this study, only 66.3% expressed a willingness to care for patients with COVID-19. This percentage is lower than that obtained in previous studies that identified students willing to respond to the significant demand for healthcare providers in the current pandemic; 74.2% of the medical and nursing students in a Spanish study [28], 71.18% of medical students in a survey across 74 countries [24], and 80% of medical students from 10 medical schools in Uganda reported that they were willing to join the medical workforce [29]. The results of the current and previous studies may be different because of the phrasing of the items and Likert response scales used for assessment, in addition to the period of data collection. Data for the previous studies were collected between March and April 2020, whereas the data for this study were collected in June 2020. Beginning in May 2020, the second wave of COVID-19 infection spread from the large cities to all the regional communities of South Korea [30]. During this time, 2.4% of the physicians and nurses were infected with COVID-19 as a result of treating patients and fulfilling other duties at the hospital [31]. The spread of COVID-19 throughout regional communities and the risk of infection posed by patients may have led to a sense of fear and anxiety in medical and nursing students [32, 33]. This may explain why more than 30% of the participants in this study were reluctant to care for patients with COVID-19.

In this study, more than half of the students (54.9%) had participated in clinical training during the pandemic, and a greater ratio of participants with clinical practice experience was found in the willingness group than in the unwillingness group. Previous studies have shown that willingness to volunteer during the COVID-19 pandemic was higher in medical students with clinical practice experience than in students without such experience [34]. Additionally, 57.8% of physicians with experience in caring for COVID-19 patients in hospitals were willing to care for patients suffering from COVID-19 [25]. It is likely that students participate both directly and indirectly in treating patients during their clinical training [34]. Therefore, healthcare providers with experience in caring for patients during disaster situations are more willing to participate in providing healthcare during future disasters because they have gained evidence-based knowledge and training in disaster management and response strategies [35]. Clinical training can help students prepare for and respond to disasters when they enter the professional workforce [36]. Moreover, since regular modes of training may not be feasible in disaster situations, educators in the healthcare field must develop new approaches to clinical training that can function even during disaster situations. For example, during the COVID-19 pandemic, the use of simulation-based virtual learning programs can be used to support students’ ongoing clinical training and evaluation [37]. This is necessary to build a healthcare workforce with the ability to respond effectively to disasters [38].

The scores for COVID-19-related knowledge, attitudes, and preventive behaviors were higher in the willingness group than in the unwillingness group. Participants’ high levels of knowledge, optimistic attitude, and preventive behaviors were correlated with their willingness to care for COVID-19 patients. Several studies have examined the relationship between students’ willingness to participate in patient care and their knowledge of infectious diseases, attitudes, and preventive behaviors [9, 16, 25, 39, 40]. The findings of the current study suggest that the following factors may increase students’ willingness to care for COVID-19 patients: understanding how COVID-19 is transmitted and how to minimize the risk of infection, maintaining infection prevention practices, and viewing government actions taken in response to the pandemic in a positive light. This study also demonstrated the relationship between students’ grade level and their willingness to care for COVID-19 patients. Senior students are at a higher educational level than sophomores and have had greater theoretical and practical educational opportunities. This may be why they showed higher willingness to care for patients with COVID-19. Additionally, the implementation of appropriate educational programs can strengthen students’ ability to manage, prepare for, and respond to disasters [36]. Attending courses on disaster preparedness and early training can enhance students’ awareness and risk perception, thereby preparing them for potential disasters [41]. However, the disaster preparedness curriculum is deficient compared with that of other public health issues; there are insufficient disaster education and training programs for medical students [36, 41]. Low levels of knowledge regarding COVID-19 due to an inadequate curriculum and lack of clinical practice opportunities, as well as negative attitudes and insufficient preventive behaviors may cause students to avoid caring for COVID-19 patients [15]. Given the positive correlation between advanced education levels and healthcare providers’ willingness to respond to disasters [35], developing a curriculum for medical and nursing students that integrates emergency response training with COVID-19-related information and practices can effectively promote their willingness to participate in patient care [42]. Educators in the field of healthcare must utilize the abundant literature on COVID-19 to help sophomore and junior students gain a deeper understanding of the pathology and epidemiology of COVID-19, as well as the treatment and care required for COVID-19 patients; this can increase students’ willingness to care for these patients.

During a pandemic such as COVID-19, a low level of willingness to participate in patient care among medical and nursing students may further burden an overloaded healthcare system, resulting in its eventual collapse. This has already been observed in many affected countries [9, 25, 35, 40]. The effect of such a collapse would be more devastating in low- and middle-income countries. To deal with a disaster situation, any healthcare workforce preparation system must have a strategic, evidence-based plan in place. Addressing the factors that contribute to the willingness of medical and nursing students, as future healthcare workers, to participate might be a key component of such planning. Therefore, the findings of this study make a valuable contribution to the development of future intervention strategies to promote medical and nursing students’ willingness to care for patients during a public health crisis.

### Limitations

There are some limitations to this study. First, as the data were collected using online questionnaires, students who do not use the Internet were excluded from this process. Second, since the data were self-reported, the study is susceptible to social desirability bias and response inaccuracy. Third, this study did not investigate the physical and mental health issues or situational factors that may be related to participants’ willingness to care for patients during the COVID-19 pandemic. Lastly, data were collected only from two regions of South Korea. Therefore, it may not be appropriate to generalize the results for the entire country. Future studies should consider various factors that may affect students’ willingness to care for patients and include a larger number of medical and nursing students from more regions of South Korea in order to obtain representative and comprehensive results.

## Conclusion

In this study, approximately two-thirds of the medical and nursing students in South Korea were willing to participate as frontline workers in the care of patients during the COVID-19 pandemic. A greater level of willingness to participate was related to the following factors: senior grade, clinical practice experience during the pandemic, higher levels of knowledge, an optimistic attitude, and preventive behaviors. The findings indicate the need to develop methods of clinical training that can be implemented even during disaster situations. Additionally, a curriculum to enhance disasters awareness must be prepared, so that students can be trained in disaster preparedness early on. Lastly, improving students’ COVID-19-related knowledge, attitudes, and preventive behaviors can lead to increased participation in patient care.

## Data Availability

The datasets generated and/or analyzed during the current study are available from the corresponding author on reasonable request. In H University, sensitive responses related to COVID-19 from students are considered as confidential information. Therefore, the data set cannot be deposited publicly.

## Abbreviations

COVID-19: Coronavirus disease 2019
SARS: Severe acute respiratory syndrome
MERS: Middle East respiratory syndrome
